# Density of Avoided Crossings and Diabatic Representation

**DOI:** 10.3390/e25050751

**Published:** 2023-05-04

**Authors:** Anatoly E. Obzhirov, Eric J. Heller

**Affiliations:** 1 Max Planck Institute for the Structure and Dynamics of Matter and Center for Free-Electron Laser Science, 22761 Hamburg, Germany; 2 Department of Physics and Chemistry and Chemical Biology, Harvard University, Cambridge, MA 02138, USA

**Keywords:** time evolution of quantum systems, structure of eigenstates and energy spectra, random matrix theory, semiclassical methods and results, atomic, molecular and solid-state systems

## Abstract

Electronic structure theory describes the properties of solids using Bloch states that correspond to highly symmetrical nuclear configurations. However, nuclear thermal motion destroys translation symmetry. Here, we describe two approaches relevant to the time evolution of electronic states in the presence of thermal fluctuations. On the one hand, the direct solution of the time-dependent Schrodinger equation for a tight-binding model reveals the diabatic nature of time evolution. On the other hand, because of random nuclear configurations, the electronic Hamiltonian falls into the class of random matrices, which have universal features in their energy spectra. In the end, we discuss combining two approaches to obtain new insights into the influence of thermal fluctuations on electronic states.

## 1. Introduction

Bloch’s theorem and translational symmetry are two of the main bricks in the foundation of solid state physics. Their use in crystal lattices makes an implicit assumption, not emphasized in the texts we are aware of. Bloch’s theorem is mathematically correct of course for a fixed, periodic potential, but real nuclei move under the influence of thermal fluctuations, so that they have random configuration without translational symmetry at any moment in time. This is clearest in the coherent state representation of the lattice, which assigns a mean position and momentum to each atom [1].

The success of solid state theory suggests there should be a physical justification for using electronic states corresponding to fixed highly symmetrical positions. Recently, it has been found that for graphene π-bands, thermal fluctuations turn out to be too fast for the adiabatic Born–Oppenheimer approximation (ABO) to be valid, so that the other limit—the diabatic limit (DBO)—holds [2].

If we define the Born–Oppenheimer approximation as supposing that the electron wavefunction returns to its starting form if the nuclei return to their starting positions after an arbitrary journey, this leaves the possibility of adiabatic and diabatic motion of the electron along the journey. We allow the caveat of possible Berry phases within the Born–Oppenheimer realm.

During thermal fluctuations, atomic positions have a random component for any arbitrary moment in time. The electronic Hamiltonian, which is a function of atomic positions, acquires this random character. Properties of simple prototype random matrix models are well studied in random matrix theory; some of them are universal and can be extended to more general cases. Here, we are interested in ensembles of parameter-dependent random matrices. In addition to level statistics, statistics of singularities in their spectrum including degeneracies and avoided crossings is known. We believe these results can be used to characterize electronic time evolution in the presence of phonons.

Indeed, the ABO breaks down when degeneracies occur; for the case of avoided crossings, Landau and Zener found that the probability of adiabatic dynamics depends on level spacing and sensitivity to perturbations. Random matrix theory combined with Landau–Zener theory makes it possible to characterize the effects of thermal fluctuations on electronic states.

The paper is organized as follows. In Section 2.1, we describe parameter-dependent random matrices; in Section 2.2 and Section 2.3, we discuss statistical properties of singularities in their spectrum, namely, conical intersections and avoided crossings. In Section 3, we describe results on graphene thermal fluctuations that suggest diabatic representation. In Section 4, we sketch future directions for describing electronic dynamics in the presence of phonons using random matrix theory.

## 2. Singularities in the Spectra of Random Matrices

### 2.1. Parameter-Dependent Random Matrices

Random matrices provide a pathway to universal behavior for complex quantum systems [3]. One example of such a system is the chaotic dynamics of a kicked top [4,5]. In this paper, we consider only Gaussian ensembles, which are denoted by the Dyson index β [3], where β=1 for GOE, β=2 for GUE, and β=4 for GSE. Corresponding matrices $H_{\beta }$ can be represented as a combination of symmetrical *S* and antisymmetrical *A* matrices, whose elements are independent Gaussian distributed random variables with mean zero and variance $(1\pm \delta_{ij})/\beta$:

(1) $$H_{1}=S,\;H_{2}=S+iA,\;H_{4}=Se_{0}+A^{1}e_{1}+A^{2}e_{2}+A^{3}e_{3}$$

where $e_{i}$ are 2 × 2 matrix representations of quaternion algebra bases:

(2) $$e_{0}=\left(\begin{array}{cc} 1 & 0\\ 0 & 1 \end{array}\right),\;e_{1}=\left(\begin{array}{cc} 0 & i\\ i & 0 \end{array}\right),\;e_{2}=\left(\begin{array}{cc} 0 & -1\\ 1 & 0 \end{array}\right),\;e_{3}=\left(\begin{array}{cc} i & 0\\ 0 & -i \end{array}\right)$$

and the direct product is assumed for $A^{n}e_{n}$.

We study the cases when a Hamiltonian $\hat{H}$ depends on a set of parameters, which, in turn, depend on time. The idea of parameter-dependent random matrices first appears in [6] to quantify the dissipation rate of a driven complex quantum system. We use the following parametrization $H(x_{1},x_{2},\cdots ,x_{n})$ suggested by Wilkinson and Austin [7]

(3) $$H\left(x_{1},x_{2},\cdots \right)=\cos x_{1}H_{1}+\sin x_{1}H_{2}+\cos x_{2}H_{3}+\sin x_{2}H_{4}+\cdots .$$

where matrices $H_{1}$, $H_{2}$, ⋯ belong to the same ensemble and $x=(x_{1},x_{2},\cdots ,x_{n})$. These matrices may also belong to different ensembles to study symmetry-breaking perturbations as Fritz Haake suggested [8,9,10].

This parametrization has a number of advantages. Distribution of matrix elements is the same for all x, and matrices *H* and $\partial H/\partial x_{j}$ are independent, therefore leading to rather simple analytical averaging within the degenerate perturbation theory; the extension to the cases when $\partial H/\partial X_{1}$ and $\partial H/\partial X_{2}$ have non-zero correlation was made by Wilkinson [11,12]. Equation (3) provides an ergodic property [13]—averaging over energy levels leads to the same level spacing distribution as averaging over parameter space for a given pair of levels—enabling, for example, geometrical considerations to determine scaling laws for the density of avoided crossings [11].

An alternative to Equation (3) is the linear parametrization

(4) $$H\left(x_{1},x_{2},\cdots \right)=H+H_{1}x_{1}+H_{2}x_{2}+\cdots .$$

However, now singularities in the spectrum happen only in the vicinity of x=0, and the distribution of eigenvalue curvatures $\partial^{2}\epsilon_{i}/\partial x_{j}^{2}$ tends to zero for $x_{j}\to \infty$.

### 2.2. Geometrical Properties of Conical Intersections

For a Hamiltonian $\hat{H}$ to have a doubly degenerate energy level, it is necessary to satisfy two independent conditions. We reproduce here the argument of Teller [14]. Let us assume that all but two of the $\hat{H}$ eigenstates have been found. Expressing $\hat{H}$ in the basis formed by these eigenstates and two arbitrary states *i* and j, forming altogether a complete set of functions, the condition for the degeneracy is

(5) $$H_{ij}=0,H_{ii}=H_{jj}.$$

For ensemble β, it leads to a system of β+1 linear equations. In two-dimensional parameter space, the levels are degenerate in isolated points called conical intersections.

The geometrical properties of conical intersections have been established in a number of papers. Longuett-Higgins found that if a wavefunction changes sign when transported adiabatically round a given loop in parameter space, then the state must become degenerate with another one at some point within the loop [15]. This topological property is one consequence of the Berry phase [16].

The distribution of conical intersections in the parameter space is characterized by density $D_{n}^{\left(\mathrm{ci}\right)}$, which is the number of points, in which energy levels *n* and n+1 are degenerate, per unit area. Wilkinson and Austin proposed the idea for its analytical derivation [11]. For a small element dA of the parameter space, the probability dp to find a conical intersection is $D_{n}^{\left(\mathrm{ci}\right)}dA$. At the same time, dp is determined by the distribution P[R] of the distances *R* from a random point to the nearest conical intersection

(6) $$dp=P\left[R\right]dR=D_{n}^{\left(\mathrm{ci}\right)}dA.$$

Hence, the problem is reduced to analytical calculation of P[R]. It is possible to express *R* by using the degenerate perturbation theory. Averaging over the corresponding ensemble leads to well-known integrals [17,18,19]. $D_{n}^{\left(\mathrm{ci}\right)}$ depends on the density of states $\rho_{n}=\rho \left(E_{n}\right)$ and the variance of the off-diagonal matrix elements $\sigma^{2}$ and has the general form $C_{\beta }{\left(\rho \sigma \right)}^{\beta +1}$:

(7) $$D_{n}^{\left(\mathrm{ci}\right)}=\frac{\pi }{3}\rho_{n}^{2}\sigma^{2},\;D_{n}^{\left(\mathrm{ci}\right)}=\frac{2\sqrt{\pi }}{3}\rho_{n}^{3}\sigma^{3},\;D_{n}^{\left(\mathrm{ci}\right)}=\frac{16\sqrt{2}\pi^{3/2}}{45}\rho_{n}^{5}\sigma^{5}\;$$

for GOE [11], GUE, and GSE [20], respectively. We note that prefactors $C_{\beta }$ were found only after corresponding scaling had been revealed in numerical calculations [21,22]. Such an order of discoveries is typical in this field, for example, the exact distribution for energy level curvatures for GUE was found only after numerical experiments [23,24,25,26].

By putting the Wigner semicircle distribution [3] in Equation (7) and integrating the resulting expression over energy *E*, one gets the density of degeneracies summed over all pairs of levels. Multiplied by the volume of the parameter space, this gives the total number of degeneracies N. For M×M GOE matrices parametrized as Equation (3), Wilkinson and Austin found [11]

(8) $$N=\frac{\pi }{2}M^{2}.$$

**Figure 1:**
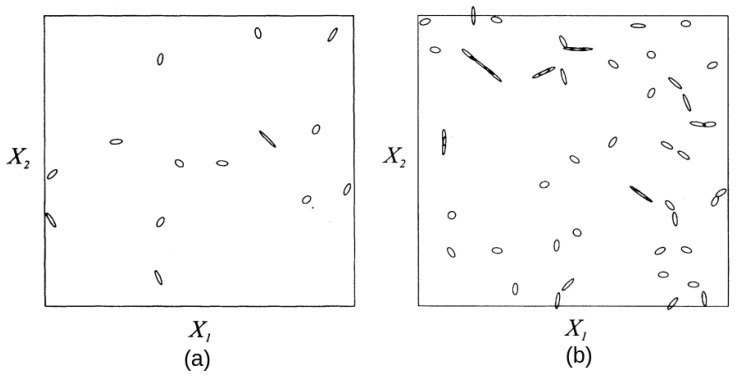
Energy level lines near the corresponding conical intersections between (**a**) the 1st and 2nd and (**b**) the 9th and the 10th energy levels for a 20×20 GOE-parametrized matrix. We note that ellipses of nearby conical intersections are correlated but still degeneracies do not exhibit repulsion. According to Equation (8), the total anticipated number of degeneracies is $\frac{\pi }{2}M^{2}=628$; in real calculations [11], it is 564. (Adapted with permission from ref. [11]. Copyright 1993 American Institute of Physics.)

For GOE-parametrized matrices, the potential energy surfaces $E_{n}\left(x_{1},x_{2}\right)$ and $E_{n+1}\left(x_{1},x_{2}\right)$ form a cone near conical intersections. The corresponding energy level lines are ellipses. Wilkinson and Austin [11] calculated the distribution of eccentricities and cone slopes. We illustrate them in Figure 1, which is taken from their paper. We note that ellipses are randomly oriented and mostly elongated in accordance with the distribution

(9) $$P\left[e\right]={\left(\frac{2e}{2-e^{2}}\right)}^{3}$$

coming from the joint probability distribution of slopes of the cone *s* and eccentricity *e*:

(10) $$P\left[t,d\right]=\frac{d}{256\sigma^{6}}e^{-t/8\sigma^{2}},$$

where *d* and *t* unambiguously determine *e* and *s* [11].

One might expect that, similar to the spacings between adjacent energy levels, conical intersections between the same pair of levels would tend to avoid each other. It turns out that this is not the case for the GOE-parametrized model, Equation (3); there, degeneracies can be considered as randomly distributed points [11]. However, as one can see on Figure 1, their eccentricities and orientations are correlated.

### 2.3. Geometrical Properties of Avoided Crossings

Consider the case when the Hamiltonian depends on only one parameter *x*. This is one of a wide range of systems with an arbitrary large number of time-dependent parameters. The noncrossing rule follows from Equation (5) [27]; when *x* is varied, two energy levels never cross. Still, they can approach each other with an arbitrary small spacing. A local minimum in the distance between adjacent energy levels is called an avoided crossing. We refer to the difference between energy levels at the minimum as a gap. The distribution of crossing gaps is known for the GOE [28,29,30], GUE, and GSE [29,30]. Surprisingly, the ratio of mean crossing to mean spacing is not universal and varies from 0.42 to 0.52 for different realizations of the same ensemble β [30]. Furthermore, known as level repulsion, the noncrossing rule has been generalized to non-hermitian Hamiltonians that describe dissipative systems [31].

Energy profiles near an avoided crossing have the hyperbolic form

(11) $$E_{n,n+1}=B\left(x-x_{0}\right)\pm \frac{1}{2}\sqrt{\epsilon^{2}+A^{2}{\left(x-x_{0}\right)}^{2}}$$

which is a solution for the Hamiltonian

(12) $$H\left(x\right)=\left(\begin{array}{cc} \left(B+A/2\right)\left(x-x_{0}\right) & \epsilon /2\\ \epsilon /2 & \left(B-A/2\right)\left(x-x_{0}\right) \end{array}\right).$$

When the Hamiltonian depends on a set of time-dependent parameters, such avoided crossings appear in the vicinity of degeneracies (the conical intersection geometry). Using this property together with ergodicity, Wilkinson and Austin have shown in a simple manner that the number of avoided crossings with gaps less than Δ is proportional to Δ in the limit Δ→0 [11].

The density of avoided crossings $D_{n}^{\left(\mathrm{ac}\right)}$ is the number of avoided crossings between *n* and n+1 energy levels per unit length as we traverse a line by varying *x*. The density $D_{\beta }^{\left(\mathrm{ac}\right)}\left(A,B,\epsilon \right)$ of avoided crossings with gap ϵ and parameters *A* and *B* of the hyperbola Equation (11) was found for β-parameterized models [6,20]

(13) $$D_{\beta }^{\left(\mathrm{ac}\right)}\left(A,B,\epsilon \right)=C_{\beta }^{\left(\mathrm{ac}\right)}\frac{\rho^{\beta +1}}{\sigma^{\beta +1}}A^{\beta +1}\epsilon^{\beta -1}\exp\left(-\frac{\beta A^{2}}{8\sigma^{2}}\right)\exp\left(-\frac{\beta B^{2}}{2\sigma^{2}}\right)\frac{\sqrt{\beta }}{\sqrt{2\pi }\sigma }dAdBd\epsilon$$

with the prefactors [20]

(14) $$C_{1}^{\left(\mathrm{ac}\right)}=\frac{\pi }{24},C_{2}^{\left(\mathrm{ac}\right)}=\frac{\pi^{3/2}}{12},C_{4}^{\left(\mathrm{ac}\right)}=\frac{8\pi^{7/2}}{135\sqrt{2}}.$$

## 3. Thermal Fluctuations in Solids

### 3.1. Born–Oppenheimer Approximation

All information about the properties of a system containing $N_{n}$ nuclei and $N_{e}$ electrons is contained in the eigenstates of the many-body Hamiltonian

(15) $$\hat{H}=\sum_{I=1}^{N_{n}}\frac{P_{I}^{2}}{2M_{I}}+\sum_{i=1}^{N_{e}}\frac{p_{i}^{2}}{2m}+\sum_{1\leq I<J}^{N_{n}}\frac{Z_{I}Z_{J}e^{2}}{|R_{I}-R_{J}|}+\sum_{1\leq i<j}^{N_{e}}\frac{e^{2}}{|r_{i}-r_{j}|}-\sum_{I=1}^{N_{n}}\sum_{i=1}^{N_{e}}\frac{Z_{I}e^{2}}{|r_{i}-R_{I}|},$$

where *m* is the mass of an electron, *e* is the charge of an electron, $r_{i}$ and $p_{i}$ are the position and the momentum of the *i*-th electron, respectively, $M_{I}$ is the mass of *I*-th nucleus, $Z_{I}$ is the charge of *I*-th nucleus, and $R_{I}$ and $P_{I}$ are the position and the momentum of *I*-th nucleus, respectively. The Hamiltonian describes kinetic energy of electrons and nuclei, Coulomb interaction between nuclei, Coulomb interaction between electrons, and finally Coulomb interaction between electrons and nuclei.

Eigenstates of Equation (15) can be found analytically only for the hydrogen atom. Numerical solutions are possible for systems containing several atoms and electrons. If there are *M* points in grids representing the positions of electrons and nuclei, $M^{3(N_{n}+N_{e})}$ real numbers would be required to store one state of the system. The exponential complexity quickly hits the memory limits of modern machines, so approximations are necessary.

The Born–Oppenheimer approximation [32,33] is the first simplification of the problem. It separates nuclear and electronic degrees of freedom. The separation is justified by the fact that nuclei are much heavier than electrons, and as a result electrons adjust to nuclear motion.

Within the Born–Oppenheimer approximation, atomic positions $R_{I}$ are fixed to give the electronic Hamiltonian

(16) $${\hat{H}}_{e}\left(R_{1},\cdots ,R_{N_{n}}\right)=\sum_{i=1}^{N_{e}}\frac{p_{i}^{2}}{2m}+\sum_{1\leq I<J}^{N_{n}}\frac{Z_{I}Z_{J}e^{2}}{|R_{I}-R_{J}|}+\sum_{1\leq i<j}^{N_{e}}\frac{e^{2}}{|r_{i}-r_{j}|}-\sum_{I=1}^{N_{n}}\sum_{i=1}^{N_{e}}\frac{Z_{I}e^{2}}{|r_{i}-R_{I}|}.$$

The electronic Hamiltonian is then diagonalized to obtain eigenvalues

(17) $${\hat{H}}_{el}\left(R_{1},\cdots ,R_{N_{n}}\right)|\psi_{n}\rangle =E_{n}\left(R_{1},\cdots ,R_{N_{n}}\right)|\psi_{n}\rangle ,$$

which are functions of nuclear positions. These functions are then regarded as potentials in which nuclei move

(18) $$H_{n}=\sum_{I=1}^{N_{n}}\frac{P_{I}^{2}}{2M_{I}}+E_{n}\left(R_{1},\cdots ,R_{N_{n}}\right).$$

This Hamiltonian includes only nuclear degrees of freedom. It can be used to obtain both quantum and classical solutions. In the classical treatment, the motion of a nuclei is approximated as

(19) $$M_{I}{\ddot{R}}_{I}=-\frac{\partial E_{n}\left(R_{1},\cdots ,R_{N_{n}}\right)}{R_{I}}.$$

In many cases, the collection of nuclei can be represented as classical harmonic oscillators, which is justified by the Schrodinger correspondence principle [34,35].

A more restrictive simplification is the clamped nuclei approximation [36], which assumes that nuclei have fixed positions corresponding to the equilibrium structure. The original problem is then reduced to finding the ground state of the electronic Hamiltonian for a fixed set of parameters, Equation (16). This problem still has exponential complexity, since $M^{3N_{e}}$ real numbers are required to store an electronic state. This complexity is overcome by a single particle picture, in which an electron moves independently of all other electrons. In this picture, the interaction of a single electron with other electrons is represented as the interaction of an electron with some average external potential due to the remaining electrons. Density functional theory provides the most accurate description of electron–electron interactions in this way [37]. Another method commonly used to solve the electronic Hamiltonian is the tight-binding method [38,39].

### 3.2. Supercell Technique

Within the Born–Oppenheimer approximation, clamped nuclei approximation, and single-particle picture, the microscopic properties of a solid can be found by solving the Schrodinger equation $\hat{H}|\psi_{n}\rangle =E_{n}|\psi_{n}\rangle$ for a single electron in the external potential set by equilibrium atomic positions $\left\{R_{I}\right\}$. The problem is still too complex, since solids consist of an infinite number of atoms and electrons, making the electronic Hamiltonian a function of an infinite number of parameters. The use of translational symmetry together with periodic boundary conditions reduces the problem to a limited number of electrons inside a primitive cell, and the Hamiltonian is hence a function of a finite set of parameters—vectors that define the primitive unit cell and atomic coordinates inside the cell. Furthermore, Bloch’s theorem gives eigenstates of the resulting Hamiltonian. Since atomic positions are fixed in this description, this solution provides only the static properties of a solid. A central property is the total energy of the system as a function of its structure. Minimizing it, one can calculate equilibrium lattice constants, the bulk modulus, and the equation of state E(V) or P(V), which is now a standard methodology in computational materials science [37].

In real crystals, of course, atoms are constantly fluctuating around equilibrium positions. At an arbitrary moment of time, atomic positions are random and there is no translational symmetry. Bloch’s theorem is no longer applicable. The problem becomes intractable since the Hamiltonian is again a function of an infinite number of parameters. In some cases, the complexity can still be harnessed by using the supercell technique [40,41]. In this technique, a supercell is constructed of *N* repeating primitive cells and the atoms inside it are allowed to move freely. Periodic boundary conditions are then imposed on this structure. It therefore becomes an infinite solid, where the supercell plays the role of a primitive cell. Translational symmetry is restored and Bloch’s theorem can be applied. The atomic motion inside the supercell allows access to dynamical properties such as phonons or relaxation times. Strictly speaking, the thus calculated dynamical properties would be different from those of the real solid; they would rather represent the properties of a large “molecule” consisting of *N* primitive cells whose motion is repeated throughout the space. However, upon an increase in *N*, the calculated properties would more closely resemble those of the real solid; in other words, by increasing *N*, it is possible to achieve numerical convergence.

Surprisingly, in many cases, the size of the supercell *N* necessary for numerical convergence is not exceedingly high in terms of numerical calculations. Physically, this means that in such cases interactions inside a solid quickly decay over distance. For example, for α-quartz, phononic dispersion can be accurately calculated by displacing atoms in a supercell consisting of only 27 unit cells. The reason is that force constants between atoms in the unit cells separated by more than three lattice spacings are weak [42]. In general, of course, the value of *N* necessary for convergence depends both on the properties and the solid, and it is possible that numerical convergence may not be achieved due to the limited computational resources of modern computers.

The dynamical properties of solids calculated using the supercell technique also rely on the assumption that electronic dynamics has a Born–Oppenheimer adiabatic character. The electronic Hamiltonian for the supercell is set by positions of the atoms inside it. Supposing the atoms are moving classically, the Hamiltonian becomes time dependent. Therefore, to describe electronic dynamics |ψ(t)〉, it is necessary to solve the time-dependent Schrodinger equation starting from the *n*-th eigenstate

(20) $$\begin{array}{cc} i\hbar \frac{\partial |\psi (t)\rangle }{\partial t} & =\hat{H}\left(t\right)|\phi (t)\rangle , \end{array}$$

(21) $$\begin{array}{cc} |\psi (0)\rangle & =|\psi_{n}\left(0\right)\rangle . \end{array}$$

The time-dependent wavefunction |ψ(t)〉 often used in supercell calculations corresponds to the *n*-th eigenstate of the Hamiltonian $\hat{H}\left(t\right)$ at time *t*

(22) $$\begin{array}{cc} \hat{H}\left(t\right)|\psi_{n}\left(t\right)\rangle & =E_{n}\left(t\right)|\psi_{n}\left(t\right)\rangle , \end{array}$$

(23) $$\begin{array}{cc} |\psi (t)\rangle & =|\psi_{n}\left(t\right)\rangle . \end{array}$$

It is different from the wavefunction that one obtains by solving the time-dependent Schrödinger equation, but according to the adiabatic theorem, they coincide if the Hamiltonian changes slowly enough (the adiabatic Born–Oppenheimer approximation (ABO)).

The adiabatic assumption can be checked by comparing the time-dependent solution with the adiabatic one. Alternatively, the adiabatic assumption can be indirectly validated by the fact that calculated dynamical observables coincide with experimental values. In most applications, there is no need for direct validation, since, for solids, theoretical predictions turn out to correctly describe the experimental behavior.

The case of graphene is unique in this regard. Graphene is a two-dimensional honeycomb structure of carbon atoms. Each carbon atom has four valence orbitals, three of which hybridize to form strong in-plane σ-bonds between adjacent atoms. The fourth electron is in a p${}_{z}$ orbital perpendicular graphene’s plane. Out-of-plane p${}_{z}$ electrons form π-bonds that are significantly weaker than σ-bonds, and therefore their impact on atomic motion is weak. Although atomic motion is primarily determined by σ-bonds, electrons in σ-bonds do not contribute to electronic transport. Therefore, the fact that numerical calculations reproduce experimental values for atomic vibrations confirms the validity of the adiabatic assumption for σ-bonds only. One can check the validity of the adiabatic theorem for π-electrons by directly comparing time-dependent electronic wavefunctions to the adiabatic ones. This comparison was performed by Mohanty and Heller [2], which we describe below.

### 3.3. Simulating Graphene Thermal Fluctuations

To model the thermal fluctuations of graphene, the authors constructed a 4 × 4 supercell with 32 carbon atoms. To check that qualitative conclusions hold for larger sizes, they repeated calculations for a 5 × 10 supercell. Within a supercell, atoms were allowed to move freely under the harmonic σ-bonded force field, with atomic motion taken as a superposition of graphene phonons that satisfied the periodic boundary conditions of a given supercell. In turn, graphene phononic frequencies were computed by considering only force constants between adjacent atoms, and the corresponding value was taken from infrared spectroscopy of the C=C aromatic bond. The amplitude of each phonon corresponded to a temperature T=300 K. Eventually, atomic positions were predetermined for each moment of time according to

(24) $$r\left(t\right)=\sum_{n}\sqrt{\frac{2k_{B}T}{M\omega_{n}^{2}}}q_{n}\cos\omega_{n}t,$$

where $q_{n}$ is the normal mode for the *n*-th phonon with frequency $\omega_{n}$. π-electrons do not directly influence this motion. This is a simplification, but it is expected that the qualitative conclusions hold since the stiff σ-bonds contribute most to the force.

At each moment of time, atomic positions determine the nearest-neighbor tight-binding Hamiltonian for π-electrons. The hopping integral between adjacent atomic sites depends on the distance $r_{ij}\left(t\right)$ between the atoms

(25) $$H\left(t\right)=-\sum_{\langle ij\rangle }\beta \exp\left[-\alpha r_{ij}\left(t\right)\right]\left({\hat{c}}_{i}^{+}{\hat{c}}_{j}+H.c.\right),$$

where 〈ij〉 stand for the indices of adjacent atoms.

The time-dependent Hamiltonian $\hat{H}\left(t\right)$ is used to obtain two types of wavefunction. The first corresponds to the adiabatic solution $|\psi_{n}\left(t\right)\rangle$, Equation (22). Adiabatic diagonalization based on the instantaneous atomic positions provides the eigenenergies $E_{n}\left(t\right)$. The second type of wavefunction is the actual electronic wavefunction that is given by Equation (20).

To study the validity of the ABO, the true wavefunction |ψ(t)〉 is compared with the adiabatic solution $|\psi_{n}\left(t\right)\rangle$ by calculating the overlap between them

(26) $$a_{n}\left(t\right)={\left|\langle \psi_{n}\left(t\right)|\psi \left(t\right)\rangle \right|}^{2},$$

which is called the adiabatic correlation.

The adiabatic correlation reveals whether the adiabatic theorem, and consequently the ABO, is valid for this process. However, it does not say how strong the changes in the wavefunctions $|\psi_{n}\left(t\right)\rangle$ and |ψ(t)〉 are. For this, the overlap of the actual wavefunction with the initial wavefunction was calculated. The corresponding quantity is called the diabatic autocorrelation

(27) $$d\left(t\right)={\left|\langle \psi (0)|\psi (t)\rangle \right|}^{2}.$$

### 3.4. Breakdown of the ABO for Thermally Fluctuating Graphene

**Figure 2:**
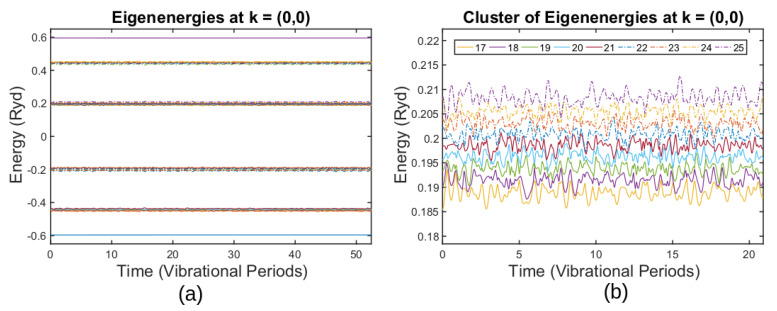
(**a**) Time-dependent energy spectrum at the Γ point. (**b**) Time evolution of the n=18th to the n=25th bands. (Reprinted from ref. [2].)

Eigenenergies of the time-dependent Hamiltonian $E_{n}\left(t\right)$ for a 4 × 4 supercell, Equation (22), are shown in Figure 2a. Time is given in the units of the shortest vibrational period, which is 191 ℏ/Ry for the case of the 4 × 4 supercell. There are 32 eigenenergies because the supercell accommodates 32 atoms; for any moment of time, eigenvalues are not degenerate because atomic positions do not correspond to a highly symmetrical configuration including the initial moment of time t=0, Equation (24). Eigenenergies form clusters that are separated from each other by gaps of several eV.

The time evolution of the eigenenergies of one of the clusters is seen to fluctuate around the average values in Figure 2b. Adjacent levels constantly approach each other; however, spacings between them never go to zero. If the dynamics were adiabatic, $E_{n}\left(t\right)$ would describe how the expected values of the time-dependent states evolve. To reveal the character of the dynamics, adiabatic correlations and diabatic autocorrelations are calculated for the states starting from the ground state and from the n=18 state at t=0.

For the ground state, the adiabatic correlation remains one $a_{1}\left(t\right)=1$ during the simulation time equal to tens of the shortest vibrational period. The diabatic autocorrelation remains one d(t)=1 as well. Preservation of adiabatic correlation means that the solution of Equation (20) |ψ(t)〉 and the solution of Equation (22) $|\psi_{1}\left(t\right)\rangle$ coincide up to an arbitrary phase, $|\psi (t)\rangle =|\psi_{1}\left(t\right)\rangle$; therefore, the adiabatic theorem and consequently the ABO hold for time evolution of this particular state. Since the diabatic autocorrelation remains one as well, we conclude that the thermal motion of atoms has no effect on the ground state, and neither the adiabatic nor the true wavefunctions change.

**Figure 3:**
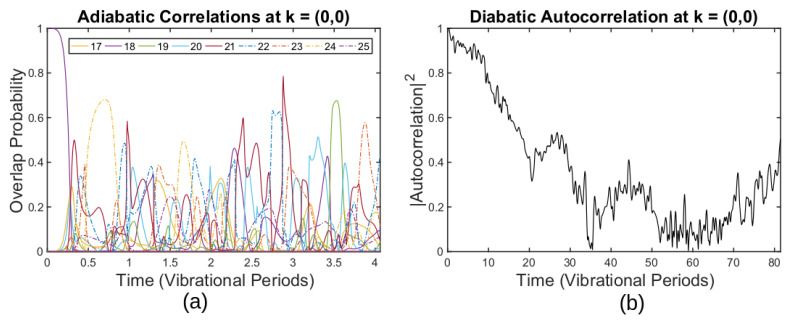
(**a**) Adiabatic autocorrelation function for the band n=18 and overlap probability between the time-dependent solution starting from the n=18th state at t=0 and other adiabatic eigenstates. (**b**) Diabatic autocorrelation function for the band n=18. (Reprinted from ref. [2].)

The behavior of higher states that form the clusters is different. Figure 3a shows adiabatic correlations for the state |ψ(t)〉, starting from the *n* = 18 eigenstate at *t* = 0. In contrast to the ground state, the overlap probability with the corresponding adiabatic state sharply decays to zero during the first vibrational period. For the first four vibrational periods, it remains zero most of the time, occasionally sharply rising to $a_{18}\approx 0.5$ and then sharply decaying to zero again three times during the aforementioned time. In addition to this, we can also observe the overlap probability with other adiabatic states in the Figure. These states form a cluster of eigenenergies, Figure 2b. The overlap with each of these states is constantly changing, rising from zero to a maximum value and then decaying back to zero. The maximum value varies among states of the cluster and lies within the range from $a_{25}\approx 0.25$ for the n=25 adiabatic eigenstate to $a_{21}=0.8$ for the *n* = 21 adiabatic eigenstate. For certain states, for example, n=21, peaks in the adiabatic correlation merge, while for the others, peaks in the adiabatic correlation stand separately.

The diabatic autocorrelation for the n=18 state also has a different behavior to the one of the ground state, Figure 3b. In contrast to the ground state case, it does not remain one throughout the simulation. It gradually decays to zero within 60 periods of the shortest vibration. The decay time of the diabatic correlation is several orders of magnitude larger than the decay time of the adiabatic correlation. After dropping to zero, the diabatic correlation function grows.

From the quick decay of the adiabatic correlations, it is clear that in the presence of thermal fluctuations, the adiabatic theorem and the ABO do not hold for states that form clusters. The adiabatic theorem and the ABO are still valid for the ground state that is separated from all the others by gaps of the order of several eV. Such a sharp decay of the adiabatic correlation can be attributed to the near degeneracy of the states, suffering frequent avoided crossings. As we discuss in the next section, these avoided crossings indeed have parameters that lead to the breakdown of the adiabatic theorem.

Secondly, the fact that the diabatic autocorrelation decays on a timescale several orders larger than adiabatic correlations means that during the very first oscillations, electronic wavefunctions stay intact in the sense that the atomic orbital coefficients remain nearly unchanged as they are carried around by their respective nuclei. Since adiabatic correlations drop sharply to zero in the meantime, one can conclude that even the smallest changes in atomic positions lead to enormous changes in the adiabatic wavefunctions $|\psi_{n}\left(t\right)\rangle$ that form a cluster and that are solutions of Equation (22). This is in sharp contrast to the ground state, where both adiabatic and diabatic wavefunctions did not change during the whole simulation time. From the perspective of the zero-th order degenerate perturbation theory, almost degenerate states form a complete basis set to describe changes in adiabatic electronic wavefunctions induced by thermal motion of atoms. Quick decay of adiabatic correlations and preservation of the initial wavefunction means that even slight atomic displacements lead to strong mixing between initial adiabatic states. This is a characteristic feature of states that form clusters.

Finally, the decay of the diabatic autocorrelation at much larger times than adiabatic correlations means that electrons cannot adjust to nuclear motion. The ABO ansatz is based on this assumption. Therefore, it is not optimal for description of such dynamical processes. Qualitatively, one may rather say that electronic states are preserved during dynamics. Since time evolution has diabatic character, then another ansatz, which the authors called the diabatic Born–Oppenheimer approximation, would be more suitable than the ABO for a description of such processes.

Qualitatively, the diabatic character is in accordance with experiments. Indeed, electronic structure theory and Bloch functions are built assuming atoms form perfect symmetrical structures. The success of electronic structure theory indicates that during thermal fluctuations, electronic wavefunctions are preserved by obeying a diabatic solution near avoided crossings. Therefore, there is an implicit diabatic assumption in electronic structure theory, otherwise it would be impossible to exploit translational symmetry. The diabatic representation is that the ABO solution for the highest symmetry is used no matter what the actual positions of the nuclei are.

## 4. Discussion and Further Work

In this section, we suggest future directions in the study of time-dependent dynamics of electrons in the presence of phonons. Mohanty and Heller [2] have established its diabatic character by running time-dependent simulations. It is interesting, however, that the lowest frequency phonons have not been studied, owing to the the finite size of the supercell. They are slow; thus, they may be again in the adiabatic realm. It would be very constructive to determine this.

We now suggest a new perspective based on avoided crossings. We outline how one can potentially come to the same conclusions by using Landau–Zener theory in tandem with random matrix theory without running time-dependent calculations.

The idea of using Landau–Zener theory in tandem with random matrix theory belongs to Wilkinson [6]. They considered a driven finite-sized system and calculated the rate of increase in energy of the system using a distribution of gap sizes and the slopes of avoided crossings, similar to Equation (13). It was noted in reference [6] that this idea could be applied in the context of the Born–Oppenheimer approximation to characterize vibronic coupling in complex molecules.

Diabatic preservation of electronic character suggests a breakdown of the adiabatic approximation. Adiabaticity breaks down when the Hamiltonian changes too rapidly. One can estimate a timescale of adiabatic motion by using Heisenberg’s uncertainty principle; for a process with the minimal spacing between energy levels ΔE, adiabatic evolution can take place only for time τ

(28) τ≥ℏ/ΔE.

From Equation (28), it follows that avoided crossings or local minima in the distance between adjacent levels determine the timescale for adiabatic motion. We have considered their statistical properties in Section 2.3.

Figure 2b shows how energy levels vary over time for graphene thermal fluctuations. Each state experiences several avoided crossings during one cycle of the shortest vibrational mode. We conclude that the presence of multiple avoided crossings is an inherent feature of this motion, and therefore should be explicitly incorporated into theoretical treatment. Specifically, avoided crossings determine a characteristic energy gap, which in turn determines a timescale for adiabatic evolution. This timescale is to be compared with the vibrational period, which is primarily set by σ-bonds and only weakly depends on p${}_{z}$-orbitals.

For the case [2], the shortest vibrational period is τ≈191ℏ/Ry, so the energy gap should be at least ΔE=1/191≈0.005 Ry. As one can see in Figure 2b, avoided crossings have a much smaller gap, leaving no hope for adiabatic motion. Indeed, in Figure 3a, the adiabatic autocorrelation of the 18th state drops to zero at t=0.25, which is exactly the first avoided crossing one can observe in Figure 2b.

Landau and Zener gave a more accurate estimation for adiabatic timescales [43]. They considered a two-level system with the Hamiltonian in the form of Equation (12) for the case $B=0,\;x_{0}=0$, and x≡t. The probability of adiabatic time evolution by the end of the motion is

(29) $$p=1-\exp\left(-\frac{\pi \epsilon^{2}}{2A\hbar }\right).$$

The estimation of adiabatic $A_{\mathrm{adia}}$ based on the exact solution $A_{\mathrm{adia}}=\frac{\pi }{2}\epsilon^{2}/\hbar$ is close to the one based on Heisenberg’s uncertainty principle, $A=\epsilon^{2}/\hbar$. In the case of graphene thermal fluctuations, the electronic structure of the p${}_{z}$ orbitals determines ϵ, while the temperature and σ-bonds determine *A*.

Thus, instead of explicitly calculating the time-dependent wavefunction, one can more simply and more insightfully study the evolution of electronic character by finding avoided crossings in the time-dependent electronic spectrum, extracting *A* and ϵ from the fit to Equation (11) and putting these parameters into Equation (29).

The procedure we have just outlined still requires performing numerical calculations to obtain a time-dependent electronic spectrum. Further analytical insights at much less effort might have been obtained using the theoretical results from Section 2.3. As a result of the randomness of thermal fluctuations, one can model the electronic Hamiltonian by a random matrix. This theory allows determining the distribution of ϵ,A, and *B* for avoided crossings, Equation (13). In tandem with Landau–Zener theory, it is possible to understand the diabatic character of time evolution even without running simulations. In order to make random matrix theory applicable, it is necessary to adjust the density of states and the sensitivity to perturbations by rescaling energy and time; this procedure has been described by Wilkinson [22].

There is one caveat: Landau–Zener theory considers only two coupled states, whereas all states that form a cluster turn out to be coupled. There is a close and suggestive analogy with the Wigner surmise for the nearest neighbor level spacing distribution; a very accurate result was obtained by Wigner using only an ensemble of 2 × 2 random matrices instead of the full N × N case.

One can see a manifestation of the full N-state cluster coupling in Figure 3a for the first avoided crossing of the 18th state; the state makes a transition to the 21st but not to the adjacent 19th or 17th state. A general solution to the multi-level Landau–Zener problem is necessary here, which has not been obtained yet [44,45,46].

We have suggested a new way to analyze electronic dynamics in the presence of phonons, which enables to use random matrix theory and may shed new light on this problem.

## 5. Summary

The main purpose of this paper is to show that two seemingly distinct subjects, random matrix theory and electronic dynamics in the presence of thermal fluctuations, can be brought together through considering statistically known properties of the electronic structure spectrum. This perspective can potentially give more information than just the preservation of diabatic electronic character recently found in the time-dependent simulations of graphene thermal fluctuations.

We first introduced parameter-dependent random matrices and the reasons for selecting a particular parametrization. Then, we considered singularities in their spectra, i.e., conical intersections and avoided crossings. We have described the geometrical properties of conical intersections, along with a typical workflow for calculating their statistical distributions analytically. Qualitatively, analytical predictions were in agreement with the illustrated numerical results. Then, we quoted the statistical distribution of fitting parameters for energy levels near an avoided crossing, which is important for the perspective we suggest.

We next examined the time evolution of graphene π-band electronic states in the presence of phonons. A direct solution of time-dependent Schrodinger equation shows that a projection of the time-dependent state on an instantaneous adiabatic state drops to zero during the very first vibrational cycle, whereas overlap with the initial electronic state decays at a much longer timescale. Moreover, it tends to recover at an even larger timescale. That indicates preservation of the electronic character and the diabatic nature of time evolution.

Finally, we sketched a possible way to treat the dynamics of electrons in the presence of phonons based on random matrix theory and avoided crossings. We started with the observation that numerous avoided crossings arise in the supercell electronic bandstructure because of nuclear motion. Overlap of the time-dependent wavefunction with the instantaneous adiabatic states suggests avoided crossings are responsible for the rapid decay of the adiabatic states. A simple estimation based on the uncertainty principle confirms that avoided crossings have gaps that are too small for the adiabatic theorem to be valid. We then outlined a more sophisticated analytical treatment based on Landau–Zener theory. All in all, the suggested perspective may enable the use of random matrix theory to characterize the time evolution of electrons in the presence of phonons. This could be a powerful new perspective in this important problem.

## Data Availability

The data presented in this study are available on request from the corresponding author.

## Abbreviations

The following abbreviations are used in this manuscript:

ABO	Adiabatic Born–Oppenheimer approximation
DBO	Diabatic Born–Oppenheimer approximation
GOE	Gaussian orthogonal ensemble
GUE	Gaussian unitary ensemble
GSE	Gaussian symplectic ensemble
